# PATIENT-REPORTED OUTCOMES AFTER PERMISSIVE WEIGHT BEARING IN SURGICALLY TREATED TRAUMA PATIENTS WITH DISPLACED INTRA-ARTICULAR CALCANEAL FRACTURES: A MULTICENTRE, RETROSPECTIVE COHORT STUDY

**DOI:** 10.2340/jrm-cc.v8.42747

**Published:** 2025-04-07

**Authors:** Coen VERSTAPPEN, Pishtiwan H.S. KALMET, Cherelle V. MADURO, Raoul VAN VUGT, Jan Bernard SINTENIE, Alexander VAN DER VEEN, Michael J.R. EDWARDS, Martijn POEZE, Erik HERMANS, Mitchell L.S. DRIESSEN

**Affiliations:** 1 Department of Trauma Surgery, Maastricht University Medical Center+, Maastricht, The Netherlands; 2 Department of Trauma Surgery, Radboud University Medical Center, Nijmegen, The Netherlands; 3 Department of Trauma Surgery, Zuyderland Medical Center, Heerlen, The Netherlands; 4 Department of Trauma Surgery, Elkerliek Hospital, Helmond, The Netherlands; 5 Department of Trauma Surgery, Catharina Hospital, Eindhoven, The Netherlands

**Keywords:** AOFAS, displaced intraarticular calcaneal fractures, trauma patients, aftertreatment, permissive weight bearing, early weight bearing

## Abstract

**Objective:**

The current aftertreatment for surgically treated patients with displaced intra-articular calcaneal fractures (DIACFs) consists of restricted weight bearing (RWB) for 8–12 weeks. This study aimed to assess whether permissive weight bearing (PWB) results in improved patient-reported outcomes (PROMs) after a minimum of 2 years follow-up, compared to RWB.

**Design:**

Multicentre, retrospective cohort study.

**Patients:**

Surgically treated patients with isolated unilateral DIACFs.

**Methods:**

Foot and ankle function was measured using the American Orthopaedic Foot and Ankle Society (AOFAS) Score and the Maryland Foot Score (MFS). Health-related quality of life was assessed using the Short Form-12 (SF-12) and the EuroQoL EQ-5D-5L (EQ-5D). Additionally, radiographic parameters and complications were recorded.

**Results:**

Fourteen patients followed the PWB and 18 followed the RWB protocol (*n* = 32). The PWB group had similar outcome scores on the AOFAS Score (83.4 vs. 71.1, *p* = 0.13) and MFS (86.3 vs. 77.6, *p* = 0.20) compared to the RWB group. PWB showed similar outcomes on the EQ-5D (0.86 vs. 0.80, *p* = 0.26) scores. Radiographic parameters and complication rates were comparable for both groups.

**Conclusion:**

This study suggests that PWB and RWB yield comparable PROMs in foot and ankle function without radiographic failures and similar complication rates in surgically treated patients with isolated, unilateral DIACFs.

## LAY ABSTRACT

This multicentre retrospective cohort study evaluated the impact of permissive weight bearing versus restricted weight bearing on patient-reported outcomes in surgically treated individuals with isolated unilateral displaced intra-articular calcaneal fractures. Functional outcomes were assessed using the American Orthopaedic Foot and Ankle Society Score and the Maryland Foot Score, while quality of life was measured via the Short Form-12 and the EuroQoL EQ-5D-5L (EQ-5D). A total of 32 patients were included, with 14 in the permissive weight bearing group and 18 in the restricted weight bearing group, after a minimum follow-up of two years. Results demonstrated no statistically significant differences between groups in functional or quality of life outcomes (e.g., American Orthopaedic Foot and Ankle Society: 83.4 vs. 71.1, *p* = 0.13; EQ-5D: 0.86 vs. 0.80, *p* = 0.26). Radiographic outcomes and complication rates were also comparable. These findings suggest that permissive weight bearing may be a safe alternative to restricted weight bearing, potentially offering greater rehabilitation flexibility without compromising clinical outcomes.

Fractures of the calcaneus account up to 2% of all fractures, 60% of the foot fractures, and approximately 65% of these are intra-articular, known as Displaced Intra-Articular Calcaneal Fractures (DIACFs) (1). These injuries are typically classified using the Sanders classification (2). Surgical intervention of calcaneal fractures is often necessary to restore the subtalar joint and the shape of the calcaneus’s shape. Given that calcaneal fractures predominantly occur in middle-aged males, the socio-economic impact is substantial (3–6). Thus, effective aftertreatment for calcaneal fractures is of utmost importance.

To enhance functional outcomes, various surgical techniques have been developed over time (7). Minimally invasive techniques, such as Percutaneous Screw Fixation (PSF), have been introduced to treat DIACFs to reduce wound complications and improve overall outcomes (8–10). The L-shaped, extensile lateral approach (ELA) remains the most commonly used method for open reduction and internal fixation (ORIF) of DIACFs (11), although the Sinus Tarsi Approach (STA) technique is increasingly accepted (12).

Currently, the *Arbeitsgemeinschaft für Osteosynthesefragen* (AO) protocol for postoperative management of DIACFs advocates non-weight bearing for up to 12 weeks, followed by partial weight bearing with a 25% increase in weight loading each week (13). This has been the standard for decades, despite the well-established benefits of early weight bearing on fracture healing and muscle and bone mass preservation (14). This raises the question of whether surgeons are overly cautious, fearing secondary fracture dislocation or mechanical construction failure.

A recent randomized controlled trial comparing early versus delayed weight bearing after operatively treated ankle fractures found early weight bearing to be clinically non-inferior and highly likely cost-effective compared delayed weight bearing (the current standard of care) (15). Additionally, a survey about the peri-operative management of calcaneal fractures revealed that 90% of surgeons do not believe there is a link between early weight bearing and complication incidence. The survey highlighted a lack of consensus on whether to restrict weight bearing for 6 or 8 to 12 weeks (16). Currently, different weight bearing protocols for calcaneal fractures are employed in hospitals in the Netherlands (17). To our knowledge, no study has compared these 2 postoperative protocols in DIACFs.

The study aimed to analyse differences in patient-reported outcomes (PROMs) (i.e. function, health-related quality of life [HRQoL]), radiographical parameters, and complications in patients with surgically treated DIACFs following either a Permissive Weight Bearing (PWB) or Restricted Weight Bearing (RWB) postoperative protocol. The hypothesis is that the results of PWB will be comparable to those of RWB, potentially demonstrating that earlier weight bearing does not compromise patient outcomes or contrarily be superior.

## PATIENTS AND METHODS

The study included surgically treated trauma patients with isolated, unilateral DIACFs, Sanders type II and III, from 2015 to 2020 across 5 hospitals in the Netherlands: Catharina Hospital, Eindhoven; Elkerliek Hospital, Helmond; Maastricht University Medical Center + (MUMC+); Radboud University Medical Center, Nijmegen (Radboudumc); and Zuyderland Medical Center, Heerlen/Sittard-Geleen. Patients were recruited between 1 May 2020 and 1 July 2022, at least 2 years after the initial trauma. Postoperatively, patients were included if they followed 1 of 2 aftertreatment protocols (PWB or RWB, respectively).

The PWB protocol allows earlier postoperative weight bearing. Progression of weight bearing is guided by the subjective experience of the patient, including factors such as pain and weight bearing tolerance. Proper patient guidance requires additional expertise on PWB, as described in the PROMETEUS protocol, from the treating physician and/or physiotherapist (18). The protocol prescribes 2 weeks of immobilization for wound healing. After this period patients are permitted to bear weight commensurate with their pain tolerance and comfort (18). For the safe use of PWB, multidisciplinary collaboration among surgeons, rehabilitative physicians, and physiotherapists is imperative. Conversely, the RWB protocol has been the standard for decades. It adheres to the AO protocol and advises non-weight bearing postoperative for up to 12 weeks, followed by partial weight bearing with a 25% increase in weight loading every week (13).

Both aftertreatment protocols (PWB and RWB) were standard of care at Zuyderland Medical Center and MUMC+. Patients treated in the other hospitals all underwent aftertreatment following the RWB protocol. All participation hospitals adhere to the same high level of trauma care and patient were treated by specialized foot and ankle surgeons.

### Inclusion criteria

Patients eligible for inclusion were aged between 18 and 80 years and needed to be able to understand and follow the instructions. Inclusion occurred after obtaining written informed consent. Exclusion criteria comprised Sanders type 4 fractures, simultaneous or existing amputation of the upper or lower limb or feet, open fractures, severe non-fracture related comorbidity of the lower extremities, pre-existent immobility, dependency in activities of daily living, rheumatoid arthritis of the lower extremities, severe psychiatric comorbidities that lead to inability to comply with the aftertreatment protocol, pathological fractures, peripheral neuropathy, alcohol or drug abuse preventing adequate follow-up, and a primary indication for arthrodesis of the subtalar joint. Moreover, all patients were considered to have a sufficient level of Dutch language.

The impact of fracture healing was carefully collected and analysed by 2 researchers (MD and CV), using patient-specific information from the Electronic Medical Registration (EMR). Data extracted from the EMR included basic patient characteristics, the American Society of Anaesthesiologists (ASA) score, trauma mechanism, and radiological parameters (2, 19).

### Primary outcome

The postoperative care adhered to standard protocols. Those willing to participate completed several questionnaires. The primary outcome measure was the function of the foot and ankle, assessed using the American Orthopaedic Foot and Ankle Society (AOFAS) Ankle-Hindfoot Score. This clinician-based score incorporates both subjective and objective information on foot and ankle function, ranging from 0 to 100, with healthy foot and ankles receiving 100 points (20, 21). Unfortunately, the minimal clinically important difference (MCID) for the AOFAS Score is unknown (22). Therefore, a difference of 10% or more in the AOFAS Score for the PWB group was estimated to be significant, reflecting a clinically relevant difference.

### Secondary outcomes

Secondary outcome measures included additional PROMs. The function of the foot and ankle was measured using the Maryland Foot Score (MFS) (23). HRQoL was assessed using the Short Form-12 (SF-12) and the EuroQoL EQ-5D-5L (EQ-5D) (24, 25).

The MFS is an assessment tool for foot disorders, consisting of items on pain, gait, functional activities, and cosmetics. It evaluates the outcomes of foot and ankle treatments and interventions. A total score of < 50 is considered poor, 50–74 fair, 75–89 good, and 90–100 excellent (23).

The SF-12 is a widely used survey for assessing HRQoL. It comprises 12 questions covering physical and mental health aspects. The scores are summarized into the Physical Component Summary (PCS) and the Mental Component Summary (MCS), reflecting overall well-being. It is a concise tool often utilized to quickly evaluate an individual’s health status, which scores ranging from 0 to 100, where higher scores indicate better well-being (24).

The EQ-5D-5L is a self-administered questionnaire containing 5 dimensions: mobility, self-care, daily activities, pain/discomfort, and depression/anxiety. Scores range from 1 (best health state) to 5 (worst health state) for each dimension, resulting in a health profile. A total score (0–1) can be calculated to facilitate comparisons with other studies (25).

Postoperative complications were defined as any complication related to the fracture that occurred during the aftertreatment regimen. These were recorded as either present or not present, along with the type of complications. Postoperative wound infections were described as defined by the US Centers for Disease Control and Prevention (CDC) and include both superficial and deep wound infections that occur after surgical procedures (26).

Superficial wound infections were described as infections occurred within 30 days after surgery and involved only the skin and subcutaneous tissue of the infection. They must meet at least one of the following criteria: purulent drainage from the superficial incision; organisms isolated from an aseptically obtained culture of fluid or tissue from the superficial incision; at least 1 sign of infection (e.g. pain or tenderness, localized swelling, redness, or heat) and the superficial incision is deliberately opened by a surgeon, unless the incision is culture negative (26).

Deep wound infections were defined as infections with an implant in place within 1 year and appear to be related to the operative procedure. These infections involve deep soft tissues (e.g. bone in calcaneal fractures) of the incision and must meet at least one of the following criteria: purulent drainage from the deep incision but not from the organ/space component of the surgical site; a deep incision spontaneously dehisces or is deliberately opened by a surgeon when the patient has at least 1 sign of infection (e.g. fever, localized pain, or tenderness), unless the incision is culture-negative; an abscess or other evidence of infection involving the deep incision found on direct examination, during reoperation, or by histopathologic or radiologic examination (26).

Routine hardware removal is not part of our standard treatment. Unlike in some other countries, hardware is only removed when causing discomfort of protruding screws. In all other cases hardware remains untouched. Therefore, hardware removal is being registered as a minor complication.

If radiological assessment data were not available in the EMR, it was performed by EH.

The medical ethics committee of Maastricht University Medical Center +, the Netherlands approved this study, and informed consent was given by all patients.

### Statistical analysis

Statistical analysis was performed using IBM SPSS Statistics, version 28.0.1 (Armonk, New York). Descriptive statistics were employed to describe the demographic data and baseline characteristics of the entire population. The Kolmogorov–Smirnov test was used to assess the normality of the data. In cases where data were not normally distributed, a Mann–Whitney U test was performed. Independent sample *t*-tests were used for normally distributed continuous data, while Chi-square tests were applied to categorical variables. Results are presented as either mean ± standard deviation (SD) or as frequencies and percentages. For non-parametric data, the median with the interquartile range (IQR) is described and a median test was used. The level of statistical significance was set at α = 0.05.

## RESULTS

### Baseline characteristics

A total of 49 patients met the inclusion criteria, of which 32 were willing to participate and completed the questionnaires, resulting in a response rate of 65.3%. The PWB group consisted of 19 patients, the RWB group of 30 patients. Patients in the PWB group were significantly older compared to those in the RWB group (56.8 years vs. 47.3 years, *p* = 0.04). No further differences in baseline characteristics or surgical techniques were found between the PWB and RWB groups. The distribution of operative techniques was equal. Characteristics of total study population are presented in Table I.

**Table I:** Baseline characteristics, total group

PROM	PWB (*n* = 19)	RWB (*n* = 30)	Total (*n* = 49)	*p*
Age at trauma (median, IQR)	60.0 (20.0)	48.5 (22.0)	53.0 (27.0)	**0.03**
Gender (% male)	84.2%	76.7%	79.6%	0.53
Preoperative Böhler’s angle (degree, SD)	10.6 (6.1)	9.0 (7.3)	9.4 (6.8)	0.62
**Sanders type**				0.10
Type II	14	15	29	
Type III	5	15	20	
ASA score (median, IQR)	2 (2)	2 (2)	2 (2)	0.98
**Type of surgery**				0.88
ORIF (ELA/STA)	58.3%	50.0%	53.6%	
PSF (Forgon-Zadravecz)	41.7%	50.0%	46.4%	

PWB: permissive weight bearing; RWB: restricted weight bearing; SD: standard deviation; ASA: American Society of Anaesthesiologists; IQR: interquartile range; ORIF: open reduction and internal fixation; ELA: extended lateral approach; STA: sinus tarsi approach; PSF: percutaneous screw fixation.

**Fig. 1:**
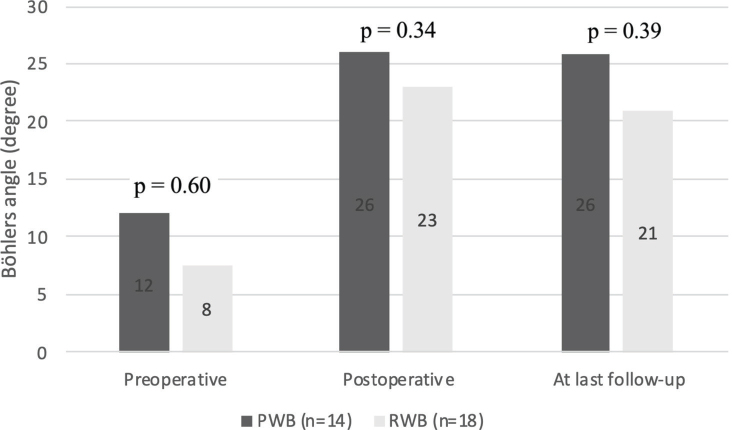
Radiological measurements.

For the patients who completed the questionnaires (*n* = 32), the preoperative, postoperative, and Böhler’s angles at last follow-up are shown in Fig. 1. No statistically significant differences in radiographic measurements were found between the 2 groups.

### Patient-reported outcome measures

The AOFAS Score was 83.4 in the PWB group compared to 71.1 in the RWB group (*p* = 0.13). In the PWB group, the MFS was 86.3 versus 77.6 in the RWB group (*p* = 0.20). The PWB group showed similar scores on the SF-12 physical component (41.4 vs. 40.8, *p* = 0.78) and the SF-12 mental component (47.4 vs. 47.8, *p* = 0.89) compared to the RWB group. The EQ-5D scored were also similar between the PWB and RWB groups (0.86 vs. 0.80, *p* = 0.26). Detailed PROM measures are shown in detail in Table II.

**Table II:** Patient-reported outcome measures

PROM	PWB (*n* = 14)	RWB (*n* = 18)	*p*
AOFAS	83.4 (14.0)	71.1 (27.1)	0.13
MFS	86.3 (9.3)	77.6 (23.2)	0.20
SF-12 physical	41.4 (5.0)	40.8 (6.2)	0.78
mental	47.4 (4.5)	47.8 (6.5)	0.89
EQ-5D	0.86 (0.13)	0.80 (0.17)	0.26

PWB: permissive weight bearing; RWB: restricted weight bearing; AOFAS: American Orthopaedic Foot and Ankle Society Ankle-Hindfoot Score; MFS: Maryland Foot Score; SF-12: Short Form-12; EQ-5D: EuroQoL EQ-5D-5L.

Smoking rates were comparable between the groups, with 28.6% in the PWB group and 33.3% in the RWB group. None of the patients used orthopaedic shoes, although several patients used insoles to improve walking: 7.1% in the PWB group and 38.9% in the RWB group.

### Complications

**Fig. 2:**
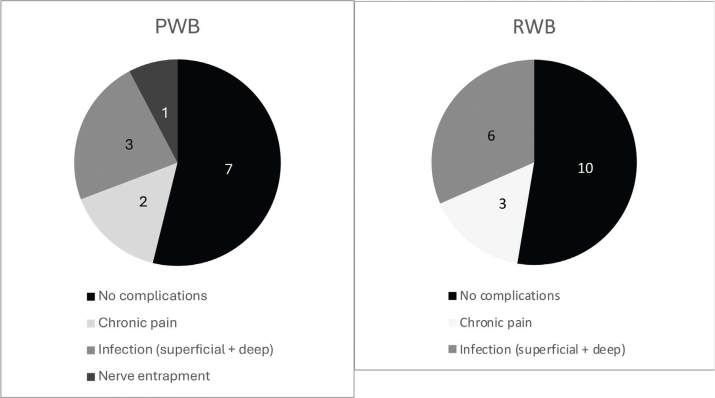
Complications (*n* = 32).

The total complication rate was not different between the RWB and PWB groups (50% vs. 55.5%). Complications in the PWB group (*n* = 14) included chronic pain (*n* = 2), infections (both superficial and deep, *n* = 3), and nerve entrapment (*n* = 1). In the RWB group (*n* = 18), complications comprised chronic pain (*n* = 3) and infections (both superficial and deep, *n* = 6). The detailed complications are shown in Fig. 2.

Reoperation rates were 42.9% in the PWB group and 27.9% in the RWB group. When comparing reoperation rates between the different surgical techniques, 26.7% of patients treated with ORIF required reoperations, whereas 53.8% of patients treated with the Forgon technique required reoperations.

## DISCUSSION

This retrospective study aimed to investigate potential differences in PROMs (i.e. function of the foot and ankle and HRQoL), radiographical parameters and postoperative complications of 2 different weight bearing protocols in the aftertreatment of surgically treated DIACFs. The result of this study suggests that PWB and RWB yield comparable outcomes results after 2 years.

As it reflects the patient’s ability to resume their normal daily life activities, recovery of the function of the foot and ankle is crucial in the management of DIACFs. The AOFAS Scores were similar between the 2 weight bearing groups. When compared to current literature in patients with RWB after DIACFs, the AOFAS score of the RWB-group of 71.1 was lower than results from a systematic review (mean AOFAS 83 points in the ‘late weight bearing’ group). However, the score for the PWB-group is comparable to the data from this review (82 points for ‘early weight bearing’) (27). This could imply that patients in RWB-group of this study had characteristics that lead to a functional outcome below average, what could be attributed to the adverse distribution of the fracture types.

To our knowledge, no data are available that directly compares the MFS for different weight bearing regimes. Nevertheless, the MFS in this study appeared to be in line with recent literature (MFS 82–90 points for different weight bearing regimes) (28–30). Although no statistically significant differences were found, both AOFAS and MFS tended to be higher in the PWB-group (a difference of 14.5 and 9.4%, respectively). Considering an increase in AOFAS Score of more than 10% as MCID for this score. It suggests that the PWB protocol could achieve comparable or even better functional outcomes.

HRQoL is another major outcome that reflects the effects of aftertreatment of DIACFs. A systematic review on the SF-12/36 in DIACFs from Alexandridis et al. found a PCS varying from 34 to 50 and a MCS between 49 and 57 points in 6 studies (31). Compared to this study, the PCS is equal and the MCS is higher. The EQ-5D in DIACFs, was compared to 2 studies. Our results showed higher scores on the EQ-5D for both PWB and RWB (32, 33). It implies that the PWB protocol may attain similar or potentially superior HRQoL.

Radiographic parameters, such as Böhler’s angle, are widely used to assess fracture reduction and alignment (34). Moreover, the postoperative Böhler’s angle is used as a variable in predicting the functional recovery. A recent study highlighted that the restoration of Böhler’s angle should be an important reduction index during surgical treatment of DIACFs (34). The results in this study showed that the postoperative Böhler’s angle was restored equally in both groups. Yet more importantly, this study population no increase in collapse of anatomically restored Böhler’s angle occurred for patients who started early weight bearing following the PWB protocol. These results are in line with a systematic review from de Boer et al. (27). In this systematic review, the postoperative Böhler angles were compared with Böhler angles at their last follow-up. Both the late and the early weight bearing group in the systematic review showed a difference of 2.5 degrees between the postoperative Böhler and the angle at their last follow-up.

Although many differences in the definition of complications exist, the complication rate was comparable in both groups and is in line with recent literature (35, 36).

To our knowledge, this study is the first study comparing PWB with RWB in isolated, unilateral DIACFs. Although this study only contains a relatively small number of patients, these findings might be the stepping stone regarding the optimal weight bearing strategy for this fracture type. While this study provides valuable insights into the outcomes of PWB and RWB in calcaneal fractures, it is important to acknowledge its *limitations*.

Firstly, as mentioned above, our relatively small number of included patients is due to the fact that displaced calcaneal fractures are a relatively rare entity. It is a fact that displaced calcaneal fractures are quite rare entity. Moreover, a significant portion of the population treated in our hospitals consists of patients who have either sustained major trauma or multiple related injuries, which restricts them from participating in our specific rehabilitation programmes. While others are not language fluently enough to participate. The latter can be accounted to the geographical location of the participating hospitals being near the German and Belgian borders.

Secondly, the fracture types of the PWB- and RWB-group differ statistically significant for the patients that filled in the questionnaires. Patients with a Sanders type II fracture were more likely to have aftertreatment following the PWB regime. This could have led to slightly distorted results favouring the PWB-group.

Moreover, it is questionable whether a follow-up of at least 2 years accurately captures the effects of an early weight bearing protocol. The results may have faded out or have been balanced over time. A study with long-term follow-up of 20 years after surgery reported that 82% of the patients experienced a clinical “steady state” 14 months postoperative (37). Moreover, a study by Kalmet et al., showed a positive effect of early weight bearing in tibial plateau fractures in the first 6 months, after which functional outcomes levelled out (38).

Another limitation was that the RWB- was available in all hospitals, while the PWB-regime was only available in 2 of the hospitals. Although there is a slight imbalance in the distribution of weight-bearing regimes, no selection bias was observed regarding the combination of surgical techniques and aftercare. Moreover, the median age was significantly lower in the RWB group. Most importantly, all participating hospitals adhere to the high standards of Dutch medical care, effectively eliminating any significant inter-hospital differences in treatment provided.

Furthermore, it can be questioned whether the comparison between the aftertreatment protocols is representative. Firstly, several studies observed an impaired compliance with weight bearing regimes of around one-third of the patients due to cognitive impairment in some of the older patients to follow instructions (39, 40). A more recent prospective comparative cohort study confirmed these data by measurements using Moticon insoles (38, 41). The study showed no significant difference in mean weight bearing between the RWB- and the PWB regime, however patients in the PWB group were bearing full weight 9 weeks earlier than those in the RWB group.

Lastly, due to the long follow-up, recall bias might have affected the results in this study. Pre-trauma level of functioning could be overestimated as times passes and the patient ages.

In conclusion, this retrospective study compared PWB and RWB in the management of surgically treated DIACFs. The results suggest that both strategies yield comparable patient reported outcome measures and a MCID in the function of the foot and ankle without radiographical failures, with comparable HRQoL and post-operative complications after 2 years. These findings imply that the choice between PWB and RWB should be tailored to individual patient characteristics, fracture patterns, and surgeons’ expertise. A randomized controlled trial is needed to comprehensively assess the efficacy and generalizability of the proposed interventions and subsequently provide guidance on evidence-based decision-making in the management of calcaneal fractures.
